# Are Nested Networks More Robust to Disturbance? A Test Using Epiphyte-Tree, Comensalistic Networks

**DOI:** 10.1371/journal.pone.0019637

**Published:** 2011-05-11

**Authors:** Martín Piazzon, Asier R. Larrinaga, Luis Santamaría

**Affiliations:** Mediterranean Institute for Advanced Studies (IMEDEA, CSIC-UIB), C/ Miquel Marquès 21, Esporles, Mallorca, Balearic Islands, Spain; Dalhousie University, Canada

## Abstract

Recent research on ecological networks suggests that mutualistic networks are more nested than antagonistic ones and, as a result, they are more robust against chains of extinctions caused by disturbances. We evaluate whether mutualistic networks are more nested than comensalistic and antagonistic networks, and whether highly nested, host-epiphyte comensalistic networks fit the prediction of high robustness against disturbance. A review of 59 networks including mutualistic, antagonistic and comensalistic relationships showed that comensalistic networks are significantly more nested than antagonistic and mutualistic networks, which did not differ between themselves. Epiphyte-host networks from old-growth forests differed from those from disturbed forest in several topological parameters based on both qualitative and quantitative matrices. Network robustness increased with network size, but the slope of this relationship varied with nestedness and connectance. Our results indicate that interaction networks show complex responses to disturbances, which influence their topology and indirectly affect their robustness against species extinctions.

## Introduction

Recent research on the architecture of mutualistic networks (e.g. plant–pollinator and plant–seed disperser [1] but also anemone-fish interactions [2] and marine cleaning symbiosis [3]) suggests that their nested structure reflects a fundamental difference from antagonistic networks, arising from how specialisation is distributed among interacting species [1], [4], [5]. In contrast to mutualistic networks, antagonistic networks (e.g., predator–prey, herbivore–plant) tend to be more compartmentalised, i.e., characterised by cohesive groups of interacting species with relatively few interactions among groups [6], [7]. Several authors have suggested that nested patterns of asymmetrical specialisation may be more likely to develop in mutualistic interactions because natural selection specifically favours the convergence and complementarity of traits in interacting species [3], [8]. In contrast, antagonistic interactions may favour greater compartmentalisation through the continual coevolution of defences and counterdefences (i.e., evolutionary arm races involving exploitation barriers), which generates greater specificity [3]. While theoretical studies have shown that the topological properties of one type of mutualistic networks (plant-pollinator) are more consistent with a mixture of complementarity and defence-counterdefence than with a predominance of complementarity [9], we are not aware of any study that has addressed the hypothesis that mutualistic networks should be more nested than non-mutualistic ones. A first step in this direction was recently made by Thébault and Fontaine [10], who showed that the nested and compartmentalised structures of mutualistic and antagonistic plant-animal interaction networks respectively maximise their persistence. However, a later commentary of their work [11] emphasizes that it does not evaluate whether differences in persistence are causing or resulting from the contrasting network architectures (i.e. “a correlation does not imply causality”). Furthermore, Gómez et al. [12] showed that phylogenetic conservatism of interaction patterns was equally likely to occur in mutualistic and antagonistic interactions, suggesting no different mechanism for both type of interactions.

In ecological networks, a nested structure indicates that reciprocal specialization is rare and, instead, specialists interact predominantly with generalists. It has been proposed that the robustness of interaction networks to anthropogenic disturbances increases with their level of nestedness, since the loss of extinction-prone specialists is less likely to trigger the extinction of other specialists in nested networks [7], [13]. To illustrate this point, Fortuna & Bascompte [14] showed that, when simulating extinctions, real-world plant-animal networks start to decay sooner but persist longer than simulated, random networks in response to habitat loss. However, no study has examined to date this hypothesis using real-world networks under different disturbance regimes. Even more, the handful of studies that have examined how mutualistic interactions respond to habitat loss or disturbance (e.g. effect of cattle ranching on pollinator networks [15]–[17]; effect of fragmentation and habitat loss on seed dispersal networks [18]–[19]) show inconclusive results. While some species proved to be very sensitive [18], others were unaffected or even benefited from disturbances [19].

Comensalistic interactions, in which one organism benefits while the other is neither helped nor harmed, provide an unexplored testing arena to understand the causes and consequences of interaction-network topology. Because neither complementarity nor defence-counterdefence traits are expected to arise in such interactions, they may provide an evolutionary model against which to evaluate mutualistic and antagonistic network properties. In particular, if the nested structure of mutualistic networks reflects the ecological effects of co-evolutionary complementarity, we would expect weaker degrees of nestedness in comensalistic networks. Moreover, should comensalistic networks prove to be nested, an evaluation of their robustness against disturbances would provide an independent test of the direct effect of network nestedness (i.e. teasing apart the potential indirect effects of trait complementarity) on its response to disturbances.

In this study, we review the existing literature on mutualistic, comensalistic and antagonistic interactions (complemented with our own data on comensalistic networks) to evaluate whether they differ in their topological properties – and, in particular, in their nestedness. We first show that comensalistic interactions are highly nested, and then use both qualitative and quantitative network analyses to evaluate their response to disturbance. For this purpose, we identify topological changes that precede rare-species extinctions (contrary to the stable network structure generally assumed by cascading-extinction simulations) and evaluate whether these changes result from neutral responses to species abundances (*sensu* Vázquez [20], i.e. “network patterns result from the fact that individuals interact randomly, so that abundant species interact more frequently and with more species than rare species”) or do also involve changes in species-specific interactions (e.g. host selectivity by epiphytes). In particular, under the hypothesis of a higher sensitivity of rare species and interactions, we expect decreased network connectedness and nestedness, and lower levels of species specialization under disturbance.

Throughout the paper, we use epiphyte-tree interactions and habitat modification/fragmentation (resulting from the logging of host trees) as model system of comensalistic networks under disturbance. Epiphyte-tree interactions can be regarded as comensalistic, since trees provide epiphytes with support for growth, releasing them from the cost of building a resistant structure, while suffering no effect from epiphyte presence [21]. We chose this model system owing to its global importance (an estimated 20,000–25,000 vascular species, representing approx. 10% of all vascular plant species, are at least occasionally epiphytic; their abundances may reach up to 50% of the local flora, and they are involved in critical ecosystem processes such as primary production, nutrient cycling, and hydrology [22]–[24]) and measurement reliability (owing to their lasting character, plant-host epiphyte networks are less vulnerable to sampling size biases introduced by the dynamic nature of most mutualistic and antagonistic networks [25]–[27]). Habitat modification and fragmentation due to logging was chosen as model disturbance owing to its global importance (it is considered as a major threat to global biodiversity [28], [29], as well as a common cause of local extinctions and even cascade co-extinctions [30]–[32]) and the well-established sensitivity of the plant-epiphyte interactions to it (since the population turnover is generally comparable for epiphytes and host trees, patch destruction and changes in host-tree dynamics caused by logging can be expected to result in direct changes in epiphyte-tree interactions; [33]).

## Materials and Methods

The study was conducted in the northeastern corner of the Chiloé Island (Chile) where, owing to the combined pressure of burn-and-clear for cattle ranching and logging for timber and firewood, once-extensive native austral forest is increasingly fragmented and disturbed [34], [35]. We selected four extensive (>300 ha) patches, two with old-growth forest (Senda Darwin, 41°53′S/73°40′W and Caulín, 41°50′S/73°36′W) and two with disturbed forest (Llanquihue, 41°51′S/73°34′W and Quilar, 41°55′S/73°36′W). Disturbed forests have been, in recent years, and are still being subjected to clear-cutting and selective logging of the largest trees. At all four patches, the most common tree species were *Drimys winteri* (Winteraceae), *Nothofagus nitida* (Fagaceae), *Tepualia stipularis* (Myrtaceae) and *Amomyrtus luma* (Myrtaceae). Differences in host trees between old-growth and disturbed forest involved mainly changes in abundance of subdominant species (e.g. increased abundance of *Raukaua laetevirens* in disturbed forest), but also a few substitutions of low-frequency species (*Azara lanceolata* and *Luma apiculata* were only found in old-growth forest, and *Raphitamnus spinosa* and *Myrceugenia parviflora* in disturbed forest).

Our surveys of tree-epiphyte networks focused on angiosperm epiphytes, including holoepiphytes (*sensu* Benzing [23]; *Sarmienta repens*), secondary hemiepiphytes (*Mirtaria coccinea, Asteranthera ovata* and *Luzuriaga polyphylla*) and the vine *Campsidium valdivianum*, but excluding facultative epiphytes (such as *Griselinia racemosa, Pernettya insana* and *Philesia magellanica)* and parasitic plants (such as the mistletoe *Tristerix corymbosus*). All forest patches studied showed the same set of epiphyte species, with the exception of one species, *Campsidium valdivianum*, which was not detected in the sampling transects of one of the old-growth forest patches (Caulín).

Hemiepiphytes were common in the low-trunk zone (<4 m), with all three groups reaching occasionally up to 15–25 m in the canopy. We conducted ground-based surveys using binoculars and, occasionally, resorting to portable ladders to confirm the identification. This method was considered reliable owing to the open structure of most tree species (low branch density), as well as the ecology (height distribution peaks at <10 m) and phenology (conspicuous flowering or fruiting during the sampling period) of most epiphyte species. Indeed, ground-based surveys carried out in forests of comparable structure at New Zealand showed high identification rates (over 90% of complete inventories) and the absence of taxonomic or ecological bias, as compared to inventories using canopy walkways [21], [36].

Owing to considerable (within-patch) spatial variation in forest composition, we also expected large variation in network structure. For this reason, we surveyed four replicate networks within each patch (placed at a minimum distance of 400 m), instead of surveying a larger number of forest patches. At each replicate site, tree-epiphyte interactions were examined along edge-centre transects (100 m long and 2 m wide). At each transect, every tree with diameter at breast height (DBH) larger than 5 cm and all angiosperm epiphytes growing on it were recorded.

Following rarefaction analysis to confirm that the number of trees sampled per transect was adequate (using EcoSim7.72 [37], [38]), we decided to analyse all interaction networks separately (i.e. considering transect-based networks as within-patch replicates reflecting spatial variation in the composition of tree-epiphyte communities). However, data were pooled into a single network per patch whenever a specific analysis did not allow for an explicit incorporation of the lack-of-independence of within-patch replicates (see below).

Firstly, we assessed plant-epiphyte network nestedness and compared it (N = 5: one network per patch, plus Burns' [21]; original data available at table S3) with a literature-based survey of mutualistic and antagonistic networks (N = 42 and 41, respectively) obtained from the NCEAS database, (http://www.nceas.ucsb.edu/interactionweb/resources.html; see full list of data sources in table S2) and Cagnolo et al. (2011). Nestedness of both observed and reviewed binary networks was estimated using two different metrics: (1) Atmar & Patterson's [39] nestedness (N hereafter), similar to the one used by Bascompte et al. [1] but calculated using an improved packing algorithm included in the BINMATNEST software [40], and (2) Almeida-Neto et al. 's [41] NODF metric, proposed as a more consistent metric of the nestedness owing to its robustness to changes in matrix shape or size. For Atmar & Patterson's N, significance was assessed against 10,000 simulations based on BINMATNEST type-3 null model (row-column probability model), while for Almeida-Neto et al. 's NODF, it was calculated for two different null models (absolute random model, Er, and row-column probability model, Ce; [1]) using the maximum number of permutations (1,000) allowed by Aninhado 3.0 software [42]. In the absolute random model, presences are randomly assigned to any cell within the matrix, while in the row-column probability model the probability that a cell *a_ij_* shows a presence is: P_ij_ = ((P_i_/C)+(P_j_/R))/2. In which P_i_ is the number of presences in the row *i*, P_j_ is the number of presences in the column *j*, C is the number of columns and R is the number of rows. Differences in nestedness among mutualistic, comensalistic and antagonistic networks were assessed in two steps: first, we corrected for the effect of network size on nestedness by fitting a reduced major axis (RMA) regression to the raw data; second, we compared the value of the residuals among the three types of networks, using one-way ANOVA followed by multiple comparisons based on Scheffé tests (Statistica 7.0).

Although previous papers (notably the seminal work by Bascompte et al. [1]) included predator-prey networks in their analyses, making the implicit assumption that they can be analyzed as two-way networks, this choice disregards the biases introduced by the repetition of certain species in both axes of the bipartite network (owing to their dual role as predator and prey) and by the fact that some of them “interact with themselves” (due to cannibalism). Hence, we decided to exclude predator-prey networks from our analysis, and based them only on 13 (plant-herbivore and parasite-host) antagonistic networks. However, to facilitate the comparison with previous work, we repeated the analyses after including predator-prey networks and present these results in Fig. S2.

Secondly, we evaluated whether plant-epiphyte networks are a direct reflection of the effect of species abundances on interaction probability (hereafter termed an “abundance effect”) by comparing the observed networks with Burns' [21] null models, where all individual epiphyte occurrences (O) from epiphyte species (*i*) were randomly assigned to a host tree species (*j*) according to the probability *P_ij_*, which was quoted to the fraction of all individual epiphyte occurrences maintained by that host species:
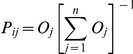



Null-model interaction matrices were constructed from the observed tree and epiphyte occurrences, and estimated quantitative matrices were turned into binary matrices by setting to 1 all cells with one or more interactions. This procedure was iterated 10,000 times for each network, using R 2.6.1 [42] (source code available on request). Congruency between the observed and expected degree of each species (numbers of links for that species) was assessed separately for trees and epiphytes, by fitting two Generalized Linear Mixed Models (GLMM; lmer function, lme4 package in R2.6.1) to observed values, with forest type (old-growth vs. disturbed) as fixed factor, individual forest patches as random factor (four replicates per patch), expected number of links as a covariate, a Poisson error distribution and a log link. A significant effect of the covariate was taken to indicate that local abundances influence the observed link frequencies, while slopes departing significantly from 1 indicate the contribution of additional processes - such as forbidden links or epiphyte preferences for certain host trees. As in all GLMs presented hereafter, we simplified the initial models (all factors and interactions) by stepwise removal of non-significant factors or interactions with P>0.20, starting by the highest-level interactions.

Thirdly, we evaluated the effects of disturbance on network topology by fitting GLMMs to several network metrics: connectance (C [44]), nestedness (N and NODF, as above), interaction strength (F [45]) and specialization/generalization index (G_k_ [17]) (see table S1 for details on the calculation of these indexes). All GLMMs included forest type (old-growth vs. disturbed) as fixed factor, forest patch as random factor, and network size (sum of rows and columns) as covariate.

Fourthly, we used a Procrustes analysis carried out with the PROTEST software [46], [47] to compare the quantitative matrices obtained in old-growth and disturbed forest patches (as in Alarcón et al. [25]). This analysis minimizes the sum-of-squares distances between corresponding observations in two matrices, by translating, reflecting, rotating and scaling one matrix to fit the other [46]. The resulting m^2^ statistic is a symmetric measure of goodness-of-fit that ranges from 0 (identical matrices) to 1 (total discordance between matrices). Its significance is evaluated against the expectation of total discordance (i.e. significant results indicate matrix concordance) by means of permutation tests (10,000 permutations per comparison, in our case), which compare one matrix to random shuffles of the other that preserve its covariance structure [46]. In addition, vector residuals obtained from the superimposition of both matrices can be used to identify the species that are responsible for the largest discrepancies between them. To meet the requirements of the method, we compared reduced forest matrices, i.e. excluding host species found only in one of the forest types used in the pairwise comparison; [46]. Comparisons using Procrustes analysis are highly sensitive to changes in species abundances; therefore, we evaluated whether discordances between matrices could be solely attributed to such changes by analysing relative-frequency matrices, in which values at each matrix cell represents the percentage of the individuals of each tree species that were occupied by each epiphyte species. Significant discrepancies between relative-frequency matrices were taken to indicate changes in “host preference” by epiphytes, i.e. increases or decreases in host-tree occupancy that are not proportional to changes in its abundance.

Finally, we evaluated whether the observed differences in network topology and/or quantitative concordance translated into changes in their robustness, in terms of sensitivity to secondary extinctions of epiphytes (resulting from simulated extinctions of host trees). We simulated host-tree extinctions using two different models: (1) “random extinctions”, where a randomly-chosen species from the extant species pool was removed at each extinction event, and (2) “rarest-species extinctions”, where the least abundant species of the extant species pool was removed at each extinction event. For each extinction event, we recorded the amount of secondary extinctions of epiphytes, (assuming that each of them becomes extinct only after loosing all its host trees in that network) and used them to estimate network robustness (R). R was defined as the area under the extinction curve (which relates the proportion of remaining host species to the proportion of extinct epiphyte species) [31] and therefore has a maximum of 1 (note the difference with the alternative method used by Dunne et al. to estimate R in food webs, which have a maximum of 0.5; [32]). The effect of forest type (old-growth vs disturbed), network topology (nestedness and connectedness) and model type (random vs. rarest-species extinctions) on robustness was subsequently analyzed using GLMMs (as above).

## Results

A total of 1360 individual trees (85 per transect, on average) belonging to 22 species were examined in our survey. Rarefaction analysis using hyperbolic functions (R^2^>0.990 in all cases) revealed that the expected numbers of links per interaction event are close to the asymptotic value for all individual networks surveyed. In order to register an extra link per network, an average of 17 trees (representing approx. 25% of sampled trees) would have to be added to each transect.

**Figure 1:**
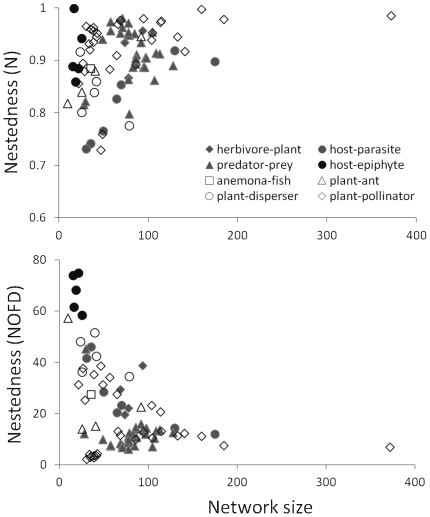
Nestedness of (epiphyte-tree) comensalistic, mutualistic and antagonistic networks. Nestedness is estimated using two different parameters: Atmar & Paterson's N [38] and Almeida-Neto's NODF [40]. Insets shows the differences in nestedness between antagonistic, comensalistic and mutualistic networks (after correction for the effect of network size: therefore “residual N” and “residual NOFD”).

Epiphyte-host tree networks were highly nested, independently of the metric used (table 1). N values ranged from 0.86 to 0.99 for the pooled matrices (forest patches), and from 0.79 to 0.99 for the individual matrices (transects). NODF values ranged from 51 to 62 (patches) and from 43 to 60 (transects). These values are particularly high in comparison to the set of mutualistic and antagonistic networks reviewed from the literature – which showed comparable or lower levels of nestedness (figure 1). After accounting for the effect of network size on N (type-2 regression: N = 0.59+0.077*logSize, F_1,57_ = 9.98, P<0.0025), differences between network types were highly significant (F_2,56_ = 8.21, P<0.0007) and pair-wise comparisons discriminated comensalistic networks from antagonistic and mutualistic ones (Scheffé-test: P<0.0009 and P<0.029, respectively), which differed marginally between themselves (P>0.052). NODF values (which, according to Almeida-Neto et al. 2008, are more robust to changes in network size) showed a comparable pattern: after accounting for the effect of network size (NODF = 110-20.7*logSize, F_1,57_ = 29.5, P<1.2*10^−6^), differences between network types were highly significant (F_2,56_ = 5.42, P<0.007), although pair-wise comparisons were only significant when comparing comensalistic and mutualistic networks (Scheffé-test: P<0.016; P>0.19 for the other two comparisons). The inclusion of predator-prey networks in the dataset did not change these results: N and NODF differed significantly among network types (F_2,85_ = 4.76, P<0.011 and F_2,85_ = 8.38, P<0.00048, respectively), because comensalistic networks were significantly more nested than antagonistic and mutualistic ones (P<0.017 for all comparisons involving comensalistic networks, P>0.17 for mutualistic vs. antagonistic ones; Fig.S1).

**Table 1:** Network properties of 16 epiphyte-tree networks measured in old-growth and disturbed forest fragments (four forest fragments, four sites per fragment).

		T	E	L	C	N	P	NODF	P(Er)	P(Ce)
**Total**		19	5	70	73.6	0.95	0.03	73.86	<0.01	0.01
**Forest Fragments:**										
**Old Growth**	Caulín	13	4	42	80.8	0.99	0.02	61.54	0.07	0.26
	Senda Darwin	11	5	37	67.3	0.89	0.22	73.94	0.02	0.09
**Disturbed**	Llanquihue	17	5	44	51.8	0.88	0.01	74.88	<0.01	0.01
	Quilar	14	5	49	70	0.86	0.25	68.13	0.13	0.32
**Individual Networks:**										
**Old Growth**	Caulín	7–10	4	13–27	46.4–67.5	0.9–0.99	1/4	67.6–68.5	3/4	2/4
	Senda Darwin	6–9	4–5	18–21	50.0–63.3	0.79–0.99	2/4	54.6–77.4	2/4	1/4
**Disturbed**	Llanquihue	5–14	4–5	10–24	22.8–55.0	0.89–0.95	1/4	66.1–79.0	2/4	1/4
	Quilar	8–11	4–5	18–28	47.5–65.6	0.89–0.93	1/4	66.1–79.0	2/4	1/4

T =  number of tree species. E =  number of epiphyte species. L =  number of links. C = network connectance. N =  network nestedness. P =  probability that the observed nestedness belongs to the distribution of null-model nestedness, based on Bascompte et al. 's [1] type 2 null model. NODF =  Almeida-Neto et al. 's nestedness metric [40], based on overlap and decreasing fill. Er =  absolute random null model. Ce =  equiproblable null model. “Forest fragment” networks are based on pooled data from its four replicate sites. For “individual networks” (replicate sites), ranges of values and the proportion of significant P-values (P<0.05) are shown.

However, the high levels of nestedness observed in comensalistic networks were largely due to an abundance effect; observed N and NODF departed from null-model estimates only in half (patches) to one-quarter (transects) of cases (table 1).

The mixed contribution of abundance-dependent and -independent effects to network topology was confirmed by Burns' null-model analysis [21], which indicated that the degree of epiphyte species is influenced, but not fully explained by (epiphyte and host tree) species abundances (see figure S1). “Expected values” was the only factor left in the reduced GLMM model, indicating a comparable effect of species abundances on epiphyte degree across all forest patches (LRT: χ^2^_1_ = 21.503, P = 3*10^−6^). However, the relationship between observed and expected values indicates that in most cases (16 out of 19) epiphytes tend to have broader degrees than predicted by the null-model; moreover, the trend is stronger for the most and least generalist species (i.e. those with the broadest and narrowest degrees).

**Figure 2:**
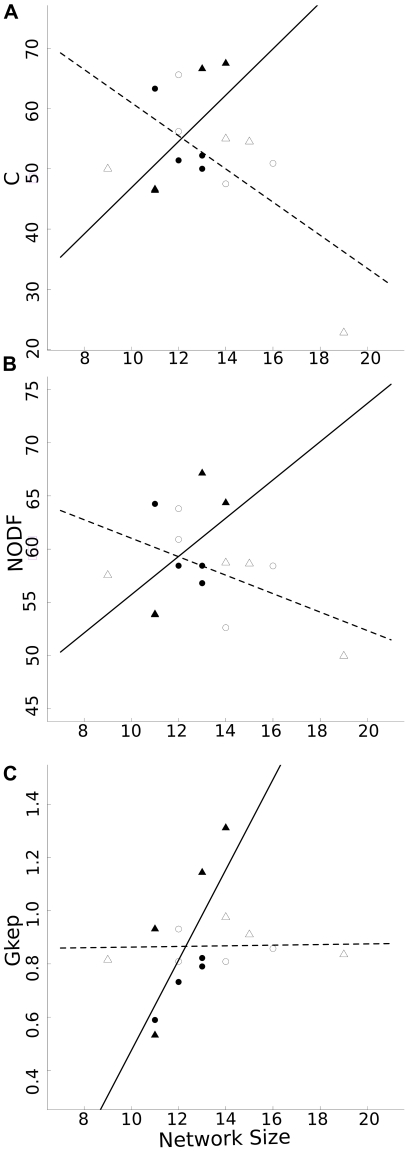
Effect of network size on several descriptors of comensalistic network topology. a) Connectance, C. b) Almeida-Neto's nestedness, NODF.[40] C) Epiphyte specialization/generalization index, G_k_^epi^. Filled symbols and solid lines indicate old-growth forest (▴ Caulin, • Senda Darwin). Empty symbols and dashed lines indicate disturbed forests (○ Quilar, Δ Llanquihue).

The results of GLMM analyses showed that plant-epiphyte networks changed their topology in response to disturbances. In three out of the six variables tested (NODF, C and G_k_^epi^) the effect of network size varied between forest types (significant “forest type * network size” interaction; table 2). Connectance and NODF increased with network size in old-growth forests, but they decreased with size in disturbed forests (figures 2a and b). Epiphyte generalization (indicated by larger values of G_k_^epi^) increased with network size in old-growth forests, but it did not vary with size in disturbed forest (figure 2c). For the three other variables (nestedness, N, strength of interaction, F, and tree specialization/generalization, G_k_^tree^), no significant effects of forest type or its interaction with network size were detected.

**Table 2:** Results of Generalized Linear Mixed Modelling evaluating the effect of network size and type of forest (old-growth vs. disturbed) on different descriptors of network topology (“dependent variable”).

Dependent variable	Type of forest	Size of Network	Type*Size
Connectance	-	-	**χ_1_^2^ = 4.88, p = 0.027**
N	χ_1_^2^ = 1.40, p = 0.24	χ_1_^2^ = 0.10, p = 0.75	-
NODF	-	-	**χ_1_^2^ = 4.04, p = 0.044**
F	χ_1_^2^ = 0.13, p = 0.72	**χ_1_^2^ = 10.0, p = 0.001**	-
G_k_ trees	χ_1_^2^ = 0.012, p = 0.91	χ_1_^2^ = 10.0, p = 0.55	-
G_k_ epiphytes	-	-	**χ_1_^2^ = 12.8, p<0.001**

Chi-square values are the results of Likelihood Ratio Tests, with their associated P-values. Figures in bold indicate significant effects (P<0.05).

Procrustes analysis (after Bonferroni correction: experiment-wise error rate = 0.05, comparison-wise error rate = 0.0083) confirmed that, as detected for qualitative networks, quantitative networks from old-growth and disturbed forest are not significantly concordant (m^2^>0.50, P>0.0083). On the other hand, comparisons within forest types indicated that forest networks were significantly concordant (old-growth: m^2^ = 0.32, P = 0.0044; disturbed: m^2^ = 0.33, P = 0.002). Residual vectors indicated that the most abundant epiphyte (*Luzuriaga poliphyla*) and tree (*Amomyrtus luma* and *Tepualia stipularis*) species generated the largest variation between networks (i.e. the greatest vector residuals for all comparisons). To evaluate whether network discordances were driven exclusively by changes in species abundances, we carried out a Procrustes analysis based on relative (instead of absolute) interaction frequencies. The results indicated a significant contribution of abundance-independent effects (old-growth and disturbed forests networks were still discordant: P>0.0083) and revealed that abundance effects actually had a homogenizing effect in old-growth networks: while disturbed forest networks were still concordant (at least marginally, P = 0.01), the concordance between old-growth forest networks disappeared (P = 0.04). As expected, residual vectors showed that the contribution of the most abundant species to matrix discordance decreased, while that from less abundant species increased.

**Figure 3:**
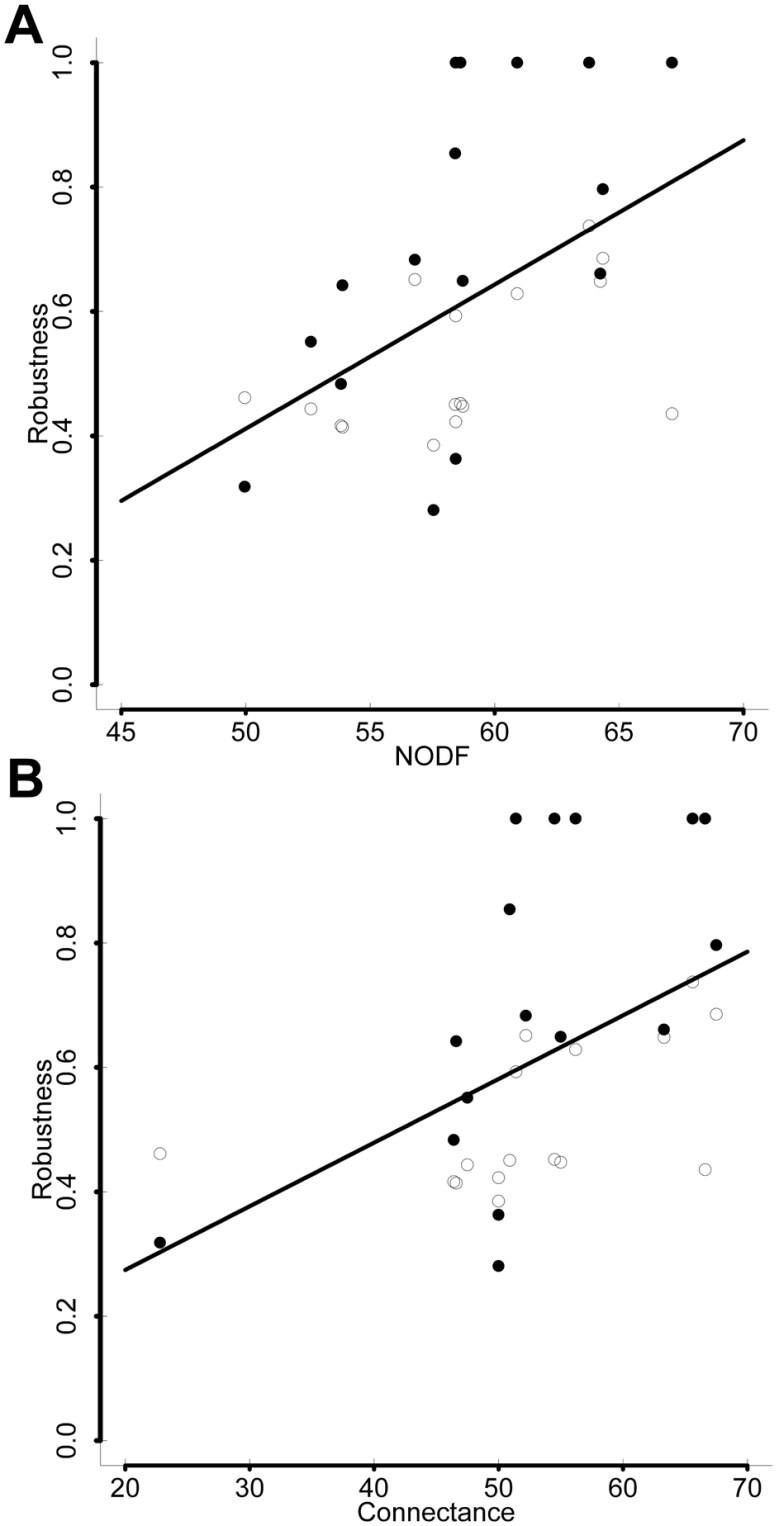
Effect of network topology on comensalistic network robustness. a) Almeida-Neto's nestedness, NODF [40], and b) connectivity, C, effect over robustness. Filled (•) and empty (○) symbols respectively indicate the results of “random” and “rarest-species” extinction simulation models.

Simulation of secondary epiphyte extinctions triggered by host-tree extinctions showed comparable results for both extinction models. While model type affected network robustness (LRT, χ^2^_1_ = 9.49, P = 0.002), which was higher for the “rarest-species” model than for the random one, none of the interactions between model type and topological factors was significant – indicating that the effect of network topology did not vary across model types. All networks were very robust to host-tree extinctions, particularly under the “rarest species model” – in which the persistence of a single tree species was generally enough to ensure the persistence of most of epiphyte species. NODF and connectance had significant, positive effects on robustness (LRT, χ^2^_1_ = 8.5 and 8.7, P = 0.003 and 0.003 respectively, Figure 3) - indicating that changes in network these topological parameters will affect network robustness. These effects did not result in significant differences in robustness between old-growth and disturbed forests. However, in the “rarest species model”, while networks from disturbed forests never lost more than one epiphyte species before removing all but the last tree species, those from primary forest loss several species in half (4/8) of the cases.

## Discussion

Our results show that (plant-epiphyte) comensalistic interactions are highly nested, particularly in comparison to the set of mutualistic and antagonistic networks reviewed from the literature (which did not differ significantly between them). The high levels of nestedness observed in comensalistic networks were, however, largely due to an abundance effect, as confirmed by the significance of the observed N and NODF values (only half to one-quarter of cases) and by Burns' null-model analysis [21]. As for the effect of disturbance on these highly-nested networks, it resulted in several topological changes that preceded rare-species extinctions and, therefore, potential extinction cascades. Connectedness, NODF and epiphyte generalization, which tended to increase with network size in old-growth forests, remained constant or decreased with size in disturbed forests. Quantitative-matrix (Procrustes) analysis confirmed both the discordance between old-growth and disturbed-forest, and the combined effect of both abundance-dependent and -independent effects thereupon. These topological changes did not have, however, a straightforward effect on network robustness, as estimated from species-extinction simulations. Robustness did not differ significantly between old-growth and disturbed forest, though it varied significantly with network size and NODF – a combination of factors shown before to vary differently in old-growth and disturbed forest.

A first, unexpected result of our analysis was that antagonistic networks did not show significantly lower nestedness than mutualistic networks. This result departed from our expectations, based on previous works (mainly Thébault & Fontaine [1], Bascompte et al. [1], and other papers that elaborated on their conclusions), of decreasing nestedness from mutualistic to commensalitic to antagonistic networks. Rather than differing from Bascompte et al. 's results [1], however, those presented here contradict their interpretation and generalizations. Bascompte et al. [1] showed that pollination and seed-dispersal networks were more nested than food-web networks, particularly after correcting for network size; their interpretation (followed by other authors, such as Guimãraes et al [3] or Ollerton et al [2]) was that this pattern can be extrapolated to mutualistic and antagonistic networks, and may be explained by their evolutionary background (development of complementary versus defence-counterdefence traits). Following this idea, Thébault & Fontaine [10] developed a population dynamic model and compared pollination and herbivore networks, and concluded that the type of interaction (mutualistic vs. antagonistic) constrains ecological networks towards different architectures. Our review focus on that interpretation and, building on recently available papers and databases, reviews a broader spectrum of networks – including anemone-fish, ant-plant and host-parasite networks. These data clearly show that antagonistic and mutualistic networks do not differ in their nestedness. It is therefore unlikely that the explanation for the nested structure of many of these networks originates in a fundamental (ecological or evolutionary) difference between mutualistic and antagonistic interactions.

A second, unexpected result was the highly nested nature of comensalistic, epiphyte-tree networks – particularly when considering their small network size. Though we cannot rule out that, given the small amount of comensalistic networks studied to date, they may prove to have comparable nestedness to mutualistic and antagonistic networks in the near future, it seems reasonable to assume that they will not be any less nested. At any rate, the high values of nestedness shown by the networks included in this study made them a perfect candidate to evaluate the effects of disturbance on network topology – thus evaluating whether the putative robustness of nested networks originates in complementary traits, supposedly characteristic of mutualistic interactions.

Our comparison of old-growth and disturbed forest networks indeed showed that, though these highly-nested networks were very robust to the strong disturbances imposed upon them (i.e. they showed small changes in species composition, despite large changes in host-tree turnover rates), they showed considerable changes in network structure and topology, which are taking place before any significant loss of epiphyte or tree species due to local extinctions. In particular, while network nestedness and connectedness increased with species richness in old-growth forests, it did the opposite in disturbed ones. This variation was largely manifested within forest patches (i.e. among transects), suggesting that while disturbed-forest communities show larger spatial variation in species richness, to the point of becoming more diverse at localized spots, they also show an impoverishment in terms of the architecture of their interactions.

Because epiphyte-tree network nestedness was caused by a combination of abundance-dependent and -independent effects, we used quantitative network (Procrustes) analysis to evaluate the relative contribution of both types of effects to the changes in network structure associated to disturbance. These analyses confirmed that the aforementioned changes were largely caused by abundance-independent effects – abundance effects having, actually, a homogenising effect in old-growth forests. The various mechanisms proposed to explain host preferences (e.g. bark peeling rate [48], water retention capacity [49], [50], host size [36], [51] or allochemical reactions [52]) are certainly worth exploring in search for more detailed causal effects behind these differences.

These findings have important bearings for all published simulation works which, assuming fixed or stable network structure, estimate the consequences of extinction chains triggered by disturbance. If network structure changes in response to disturbance, these changes must be understood and incorporated to such simulations. To evaluate the potential influence of the observed changes in network structure on robustness estimates, we performed a simple extinction-chain analysis based on the networks observed in old-growth and disturbed forest. The results indicate that, though the direct effects of disturbance on robustness (in terms of differences between old-growth and disturbed forests) are of limited importance, it may have significant indirect effects mediated by changes in network topology (since network robustness increased with both nestedness and connectance).

Owing to the complex interactions between disturbance, network size, NODF and C, estimating the outcome of forest disturbance of plant-epiphyte networks will require more extensive surveys and simulations. However, a first estimate indicates that, in comparison with disturbed forests, old-growth forests will be particularly sensitive to spatial or inter-patch variation in network size. In these forests, local increases in network size will result in increasing nestedness and connectance, which will in turn result in increased robustness. In contrast, disturbed forest will show the opposite effect: increased network size results in decreased NODF and connectance, which in turn result in decreased robustness. The net result is therefore that old-growth patches (or sites within patches) with few species will be less robust to extinctions than disturbed patches (or sites), while those with many species will be more robust than disturbed patches (or sites).

Old-growth forests can therefore be predicted to depend on the preservation of species-rich patches for the maintenance of the architecture of their interactions; while, in disturbed forests, all sites or patches will be roughly equivalent. Our analysis thus stresses the importance of spatial heterogeneity to understand key aspects of community structure and dynamics even in cases, such as network analysis, where spatial relationships tend to be explicitly ignored.

## Supporting Information

Figure S1 **Relationship between observed epiphyte-species**' **degrees and those predicted by Burn**'**s (2007) null model**. Fitted exponential line represents the best fit for the data (y = exp(1.20+0.11*x), R2 = 0.98).
(DOC)

Figure S2 **Nestedness of comensalistic, mutualistic and antagonistic networks.** Nestedness is estimated using two different parameters: Atmar & Paterson's N [38] and Almeida-Neto's NODF [40]. Insets shows the differences in nestedness between antagonistic, comensalistic and mutualistic networks (after correction for the effect of network size: therefore “residual N” and “residual NOFD”).
(DOC)

Table S1 **Details on the calculation of used indexes.**
(DOC)

Table S2 **Data sources for all networks included in the analyses.** Most of them are available at the NCEAS database (http://www.nceas.ucsb.edu/interactionweb/resources.html).
(DOC)

Table S3 **Epiphyte-host tree quantitative networks sampled during the field survey**. a) and b) are old-growth forests, while c) and d) are disturbed ones.
(DOC)
