# Supplementary material for: Are Nested Networks More Robust to Disturbance? A Test Using Epiphyte-Tree, Comensalistic Networks
Source: PLoS One. 2011 May 11;6(5):e19637. doi: 10.1371/journal.pone.0019637 (PMC3092765; doi:10.1371/journal.pone.0019637)
Supplement: Figure S1 — Relationship between observed epiphyte-species' degrees and those predicted by Burn's (2007) null model. Fitted exponential line represents the best fit for the data (y = exp(1.20+0.11*x), R2 = 0.98). (DOC) [file pone.0019637.s001.doc]

Figure S1.
