# Supplementary figures and images for: Are Nested Networks More Robust to Disturbance? A Test Using Epiphyte-Tree, Comensalistic Networks
Source: PLoS One. 2011 May 11;6(5):e19637. doi: 10.1371/journal.pone.0019637 (PMC3092765; doi:10.1371/journal.pone.0019637)

Figure S2.


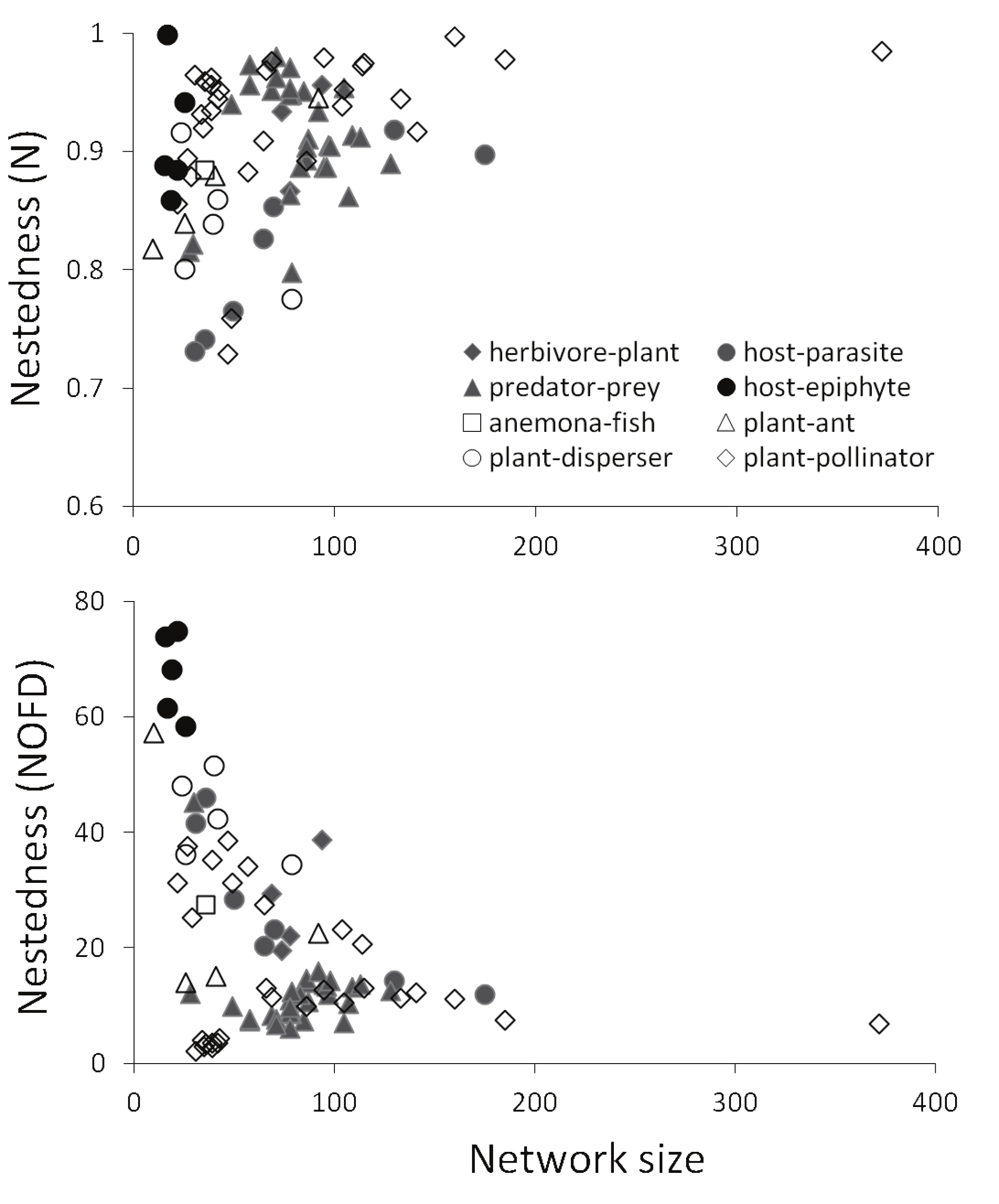

Supplement: Figure S2 — Nestedness of comensalistic, mutualistic and antagonistic networks. Nestedness is estimated using two different parameters: Atmar & Paterson's N [38] and Almeida-Neto's NODF [40]. Insets shows the differences in nestedness between antagonistic, comensalistic and mutualistic networks (after correction for the effect of network size: therefore “residual N” and “residual NOFD”). (DOC) [file pone.0019637.s002.doc]
