# Supplementary material for: Are Nested Networks More Robust to Disturbance? A Test Using Epiphyte-Tree, Comensalistic Networks
Source: PLoS One. 2011 May 11;6(5):e19637. doi: 10.1371/journal.pone.0019637 (PMC3092765; doi:10.1371/journal.pone.0019637)
Supplement: Table S1 — Details on the calculation of used indexes. (DOC) [file pone.0019637.s003.doc]

Table S1.

**Index name Calculation Reference**

Connectance(*C*) C=L/(T+E) Jordano, 1987

**Details:** Being *L* the total number of links detected and *T* and *E* the number of tree and epiphyte species respectively

Interaction strength (*F*) $F=d_{ij}^{E}\cdot d_{ji}^{T}$ Aizen et al., 2008

**Details:** $d_{ij}^{T}$ is the fraction of all epiphytes from one species colonizing a particular tree species and $d_{ij}^{E}$ is the fraction of each

tree species which have one particular epiphytes species

Specialization/generalization index for species (*G_i_*) $E_{i}=\frac{-\sum_{j} p_{j}\cdot\text{ln}p_{j}}{\text{ln}S_{i}}$ Taki & Kevan, 2007

$$R_{i}=\frac{S_{i}}{N}$$

$$G_{i}=R_{i}\cdot E_{i}$$

**Details:** *S_i_* is the number of partner species interacting with species *i*, and *N* is the total number of species on the partners’ side

of the community. and *p_j_* is the proportion of individuals corresponding to the *jth* partner species of the given species *i*.

Specialization/generalization index for community (*C_k_*) $C_{k}=\sum_{i}^{D} \frac{G_{i}\cdot t_{i}}{t_{D}}$ Taki & Kevan, 2007

**Details:** *D* is the total number of species in community *k,* $t_{i}$is the total number of individuals of species *I and* *t_D_* is the total number of individuals in community *k*.
