# Supplementary material for: Are Nested Networks More Robust to Disturbance? A Test Using Epiphyte-Tree, Comensalistic Networks
Source: PLoS One. 2011 May 11;6(5):e19637. doi: 10.1371/journal.pone.0019637 (PMC3092765; doi:10.1371/journal.pone.0019637)
Supplement: Table S2 — Data sources for all networks included in the analyses. Most of them are available at the NCEAS database (http://www.nceas.ucsb.edu/interactionweb/resources.html). (DOC) [file pone.0019637.s004.doc]

Table S2.

| **Type of network** | **Classification** | **Number of networks** | **References** |
| --- | --- | --- | --- |
| Hervibore-plant | antagonistic | 2 | Joern, A. 1979. Feeding patterns in grasshoppers (Orthoptera: Acrididae): factors influencing diet specialization. Oecologia 38: 325-347. |
| Hervibore-plant | antagonistic | 1 | Cagnolo, L., Salvo, A. and Valladares, G. 2011. Network topology: patterns and mechanisms in plant-herbivore and host-parasitoid food webs. Journal of Animal Ecology, 80, 342-351. |
| Hervibore-plant | antagonistic | 2 | Leather, S.R. 1991. Feeding specialisation and host distribution of British and Finnish Prunus feeding macrolepidoptera. OIKOS 60: 40-48. |
| Host-parasite | antagonistic | 7 | Arai, H. P., and D. R. Mudry. 1983. Protozoan and metazoan parasites of fishes from the headwaters of the Parsnip and McGregor Rivers, British Columbia: a study of possible parasite transfaunations. Canadian Journal of Fisheries and Aquatic Sciences 40:1676-1684.  Arthur, J. R., L. Margolis, and H. P. Arai. 1976. Parasites of fishes of Aishihik and Stevens Lakes, Yukon Territory, and potential consequences of their interlake transfer through a proposed water diversion for hydroelectrical purposes. Journal of the Fisheries Research Board of Canada 33:2489-2499.  Bangham, R. V. 1955. Studies on fish parasites of Lake Huron and Manitoulin Island. American Midland Naturalist 53:184-194.  Chinniah, V. C., and W. Threlfall. 1978. Metazoan parasites of fish from the Smallwood Reservoir, Labrador, Canada. Journal of Fish Biology 13:203-213.  Dechtiar, A. O. 1972. Parasites of fish from Lake of the Woods, Ontario. Journal of Fisheries Research Board of Canada 29:275-283.  Leong, T. S., and J. C. Holmes. 1981. Communities of metazoan parasites in open water fishes of Cold Lake, Alberta. Journal of Fish Biology 18:693-713. |

| Host-parasite | Antagonistic | 1 | Cagnolo, L., Salvo, A. and Valladares, G. 2011. Network topology: patterns and mechanisms in plant-herbivore and host-parasitoid food webs. Journal of Animal Ecology, 80, 342-351. |
| --- | --- | --- | --- |
| Predator-prey | antagonistic | 1 | Lafferty, K. D., Hechinger, R. F., Shaw, J. C., Whitney, K. L. & Kuris, A. M. (2006b) Food webs and parasites in a salt marsh ecosystem. In Disease ecology: community structure and pathogen dynamics (ed. S. Collinge & C. Ray), pp. 119-134. Oxford: Oxford University Press. |
| Predator-prey | antagonistic | 26 | Jaarsma, N.G., de Boer, S.M., Townsend, C.R., Thompson, R.M. and Edwards, E.D. 1998. Characterising food webs in two New Zealand streams. New Zealand Journal of Marine and Freshwater Research 32: 271-286.  Thompson, R.M., Edwards, E.D., McIntosh, A.R. and Townsend, C.R. 2001. Allocation of effort in stream food web studies â€“ the best compromise? Marine and Freshwater Research 52/3: 339-345.  Thompson, R.M. and Townsend, C.R. 1999. The effect of seasonal variation on the community structure and food-web attributes of two streams : implications for food-web science. Oikos 87: 75-88.  Thompson, R.M. and Townsend, C.R. 2000. Is resolution the solution? the effect of taxonomic resolution on the calculated properties of three stream food webs. Freshwater Biology 43: 1-10.  Thompson, R.M. and Townsend, C.R. 2003. Impacts on stream food webs of native and exotic forest: an intercontinental comparison. Ecology 84: 145-161.  Thompson, R.M. and Townsend, C.R. In press. Energy availability, spatial heterogeneity and ecosystem size predict food-web structure in streams. Oikos.  Townsend, C.R., Thompson, R.M., McIntosh, A.R., Kilroy, C., Edwards, E.D. and Scarsbrook, M.R. 1998. Disturbance, resource supply and food-web architecture in streams. Ecology Letters 1: 200-209. |
| Host-epiphyte | comensalistic | 1 | Burns KC (2007) Network properties of an epiphyte metacommunity J. Ecol. 95: 1142-1151. |

| Anemona-fish | mutualistic | 1 | Ollerton, J., McCollin, D., Fautin, D.G. & Allen, G.R. (2007) Finding NEMO: nestedness engendered by mutualistic organisation in anemonefish and their hosts. Proceedings of the Royal Society B, doi:10.1098/rspb.2006.3758. |
| --- | --- | --- | --- |
| Plant-ant | mutualistic | 1 | Blüthgen, N., N. E. Stork, and K. Fiedler. 2004. Bottom-up control and co-occurrence in complex communities: honeydew and nectar determine a rainforest ant mosaic. Oikos 106: 344-358. |
| Plant-ant | mutualistic | 1 | Davidson, D.W., R.R. Snelling, and J.T. Longino. 1989. Competition among ants for myrmecophytes and the significance of plant trichomes. Biotropica 21: 64-73. |
| Plant-ant | mutualistic | 1 | Davidson, D.W., and B.L. Fisher. 1991. Symbiosis of ants with Cecropia as a function of light regime. Pages 289-309 in C.R. Huxley and D.F. Cutler, eds., Ant-Plant Interactions. Oxford University Press, Oxford. |
| Plant-ant | mutualistic | 1 | Fonseca, C.R., and G. Ganade. 1996. Asymmetries, compartments and null interactions in an Amazonian ant-plant community. Journal of Animal Ecology 66: 339-347. |
| Plant-disperser | mutualistic | 1 | Beehler, B. 1983. Frugivory and polygamy in birds of paradise. The Auk 100: 1-12. |
| Plant-disperser | mutualistic | 1 | Poulin, Brigitte, S. Joseph Wright, Gaetan Lefebvre, and Osvaldo Calderon (1999) Interspecific synchrony and asynchrony in the fruiting phenologies of congeneric bird-dispersed plants in Panama. Journal of Tropical Ecology 15: 213-227. |
| Plant-disperser | mutualistic | 1 | Snow, B.K. and D.W. Snow. 1971. The feeding ecology of Tanagers and honeycreeperes in Trinidad. The Auk 88: 291-322 |
| Plant-disperser | mutualistic | 1 | Snow, B.K. and D.W. Snow. 1988. Birds and Berries. Calton, England. |
| Plant-disperser | mutualistic | 1 | Sorensen, A.E. 1981. Interactions between birds and fruit in a temperate woodland. Oecologia 50: 242-249. |
| Plant-pollinator | mutualistic | 3 | Arroyo, M. T. K., R. B. Primack, and J. J. Armesto. 1982. Community studies in pollination ecology in the high temperate Andes of Central Chile. I. Pollination mechanisms and altitudinal variation. American Journal of Botany 69:82-97. |
| Plant-pollinator | mutualistic | 1 | Barrett, S. C. H., and K. Helenurm. 1987. The Reproductive-Biology of Boreal Forest Herbs.1. Breeding Systems and Pollination. Canadian Journal of Botany 65:2036-2046. |

| Plant-pollinator | mutualistic | 1 | Clements, R. E., and F. L. Long. 1923, Experimental pollination. An outline of the ecology of flowers and insects. Washington, D.C., USA, Carnegie Institute of Washington. |
| --- | --- | --- | --- |
| Plant-pollinator | mutualistic | 1 | Dupont YL, Hansen DM and Olesen JM (2003) Structure of a plant-flower-visitor network in the high-altitude sub-alpine desert of Tenerife, Canary Islands. Ecography 26:301-310. |
| Plant-pollinator | mutualistic | 1 | Elberling, H., and J. M. Olesen. 1999. The structure of a high latitude plant-flower visitor system: the dominance of flies. Ecography 22:314-323. |
| Plant-pollinator | mutualistic | 1 | Hocking, B. 1968. Insect-flower associations in the high Arctic with special reference to nectar. Oikos 19:359-388. |
| Plant-pollinator | mutualistic | 1 | Inouye, D. W., and G. H. Pyke. 1988. Pollination biology in the Snowy Mountains of Australia: comparisons with montane Colorado, USA. Australian Journal of Ecology 13:191-210. |
| Plant-pollinator | mutualistic | 1 | Kato, M., T. Makutani, T. Inoue, and T. Itino. 1990. Insect-flower relationship in the primary beech forest of Ashu, Kyoto: an overview of the flowering phenology and seasonal pattern of insect visits. Contr. Biol. Lab. Kyoto Univ. 27:309-375. |
| Plant-pollinator | mutualistic | 1 | McCullen, C. K. 1993. Flower-visiting insects of the Galapagos Islands. Pan-Pacific Entomologist 69:95-106. |
| Plant-pollinator | mutualistic | 2 | Medan, D., N. H. Montaldo, M. Devoto, A. Mantese, V. Vasellati, and N. H. Bartoloni. 2002. Plant-pollinator relationships at two altitudes in the Andes of Mendoza, Argentina. Arctic Antarctic and Alpine Research 34:233-241. |
| Plant-pollinator | mutualistic | 1 | Memmott, J. 1999. The structure of a plant-pollinator food web. Ecology Letters 2:276-280. |
| Plant-pollinator | mutualistic | 1 | Mosquin, T., and J. E. H. Martin. 1967. Observations on the pollination biology of plants on Melville Island, N.W.T., Canada. Canadian Field Naturalist 81:201-205. |
| Plant-pollinator | mutualistic | 1 | Motten, A. F. 1982. Pollination Ecology of the Spring Wildflower Community in the Deciduous Forests of Piedmont North Carolina. Doctoral Dissertation thesis, Duke University, Duhram, North Carolina, USA.  Motten, A. F. 1986. Pollination ecology of the spring wildflower community of a temperate deciduous forest. Ecological Monographs 56:21-42. |

| Plant-pollinator | mutualistic | 2 | Olesen, J. M., L. I. Eskildsen, and S. Venkatasamy. 2002. Invasion of pollination networks on oceanic islands: importance of invader complexes and endemic super generalists. Diversity and Distributions 8:181-192. |
| --- | --- | --- | --- |
| Plant-pollinator | mutualistic | 1 | Ollerton, J., S. D. Johnson, L. Cranmer, and S. Kellie. 2003. The pollination ecology of an assemblage of grassland asclepiads in South Africa. Annals of Botany 92:807-834. |
| Plant-pollinator | mutualistic | 1 | Ramirez, N., and Y. Brito. 1992. Pollination Biology in a Palm Swamp Community in the Venezuelan Central Plains. Botanical Journal of the Linnean Society 110:277-302. |
| Plant-pollinator | mutualistic | 1 | Robertson, C. 1929. Flowers and insects: lists of visitors to four hundred and fifty-three flowers. Carlinville, IL, USA, C. Robertson. |
| Plant-pollinator | mutualistic | 1 | Schemske, D. W., M. F. Willson, M. N. Melampy, L. J. Miller, L. Verner, K. M. Schemske, and L. B. Best. 1978. Flowering Ecology of Some Spring Woodland Herbs. Ecology 59:351-366. |
| Plant-pollinator | mutualistic | 1 | Small, E. 1976. Insect pollinators of the Mer Bleue peat bog of Ottawa. Canadian Field Naturalist 90:22-28. |
| Plant-pollinator | mutualistic | 8 | Vázquez, D. P. 2002. Interactions among Introduced Ungulates, Plants, and Pollinators: A Field Study in the Temperate Forest of the Southern Andes. Doctoral Dissertation thesis, University of Tennessee, Knoxville, Tennessee, USA.  Vázquez, D. P., and D. Simberloff. 2002. Ecological specialization and susceptibility to disturbance: conjectures and refutations. American Naturalist 159:606-623.  Vázquez, D. P., and D. Simberloff. 2003. Changes in interaction biodiversity induced by an introduced ungulate. Ecology Letters 6: 1077-1083. |
