# Supplementary material for: Are Nested Networks More Robust to Disturbance? A Test Using Epiphyte-Tree, Comensalistic Networks
Source: PLoS One. 2011 May 11;6(5):e19637. doi: 10.1371/journal.pone.0019637 (PMC3092765; doi:10.1371/journal.pone.0019637)
Supplement: Table S3 — Epiphyte-host tree quantitative networks sampled during the field survey. a) and b) are old-growth forests, while c) and d) are disturbed ones. (DOC) [file pone.0019637.s005.doc]

Table S3.

1. CAULIN FOREST

|  |  | Epiphyte species | | | |
| --- | --- | --- | --- | --- | --- |
|  |  | *Miraria coccinea* | *Asteranthera ovata* | *Luzuriaga polyphylla* | *Sarmienta repens* |
| Tree species | *Gevuina avellana* | 7 | 0 | 15 | 0 |
|  | *Drymis winteri* | 2 | 5 | 29 | 1 |
|  | *Crinodendron hookerianum* | 0 | 0 | 4 | 0 |
|  | *Nothofagus dombeyi* | 3 | 7 | 27 | 3 |
|  | *Amomyrtus luma* | 2 | 2 | 17 | 2 |
|  | *Saxegothaea conspicua* | 6 | 1 | 12 | 2 |
|  | *Podocarpus nubigenus* | 1 | 1 | 4 | 1 |
|  | *Amomyrtus meli* | 3 | 0 | 2 | 1 |
|  | *Laureliopsis phillippiana* | 7 | 2 | 14 | 5 |
|  | *Tepualia stipularis* | 8 | 10 | 25 | 1 |
|  | *Caldcluvia paniculata* | 3 | 2 | 16 | 2 |
|  | *Weinmannia trichosperma* | 0 | 0 | 1 | 0 |
|  | *Eucryphia cordifolia* | 4 | 0 | 8 | 2 |

1. SENDA DARWIN FOREST

|  |  | Epiphyte species | | | | |
| --- | --- | --- | --- | --- | --- | --- |
|  |  | *Miraria coccinea* | *Asteranthera ovata* | *Luzuriaga polyphylla* | *Sarmienta repens* | *Campsidium valdivianum* |
| Tree species | *Azara lanceolata* | 1 | 1 | 4 | 0 | 0 |
|  | *Luma apiculata* | 4 | 0 | 11 | 1 | 0 |
|  | *Gevuina avellana* | 0 | 1 | 1 | 0 | 0 |
|  | *Drymis winteri* | 14 | 2 | 61 | 0 | 20 |
|  | *Nothofagus dombeyi* | 14 | 3 | 32 | 0 | 11 |
|  | *Amomyrtus luma* | 3 | 0 | 8 | 0 | 0 |
|  | *Amomyrtus meli* | 2 | 0 | 1 | 2 | 1 |
|  | *Myrceugenia planipes* | 0 | 0 | 2 | 1 | 0 |
|  | *Tepualia stipularis* | 8 | 5 | 33 | 3 | 6 |
|  | *Caldcluvia paniculata* | 8 | 2 | 7 | 1 | 1 |
|  | *Eucryphia cordifolia* | 2 | 1 | 6 | 0 | 0 |

1. LLANQUIHUE FOREST

|  |  | Epiphyte species | | | | |
| --- | --- | --- | --- | --- | --- | --- |
|  |  | *Miraria coccinea* | *Asteranthera ovata* | *Luzuriaga polyphylla* | *Sarmienta repens* | *Campsidium valdivianum* |
| Tree species | *Gevuina avellana* | 3 | 0 | 2 | 0 | 0 |
|  | *Drymis winteri* | 24 | 9 | 85 | 1 | 3 |
|  | *Crinodendron hookerianum* | 1 | 0 | 2 | 0 | 0 |
|  | *Nothofagus dombeyi* | 13 | 1 | 26 | 0 | 1 |
|  | *Amomyrtus luma* | 20 | 5 | 62 | 2 | 6 |
|  | *Saxegothaea conspicua* | 3 | 1 | 6 | 0 | 0 |
|  | *Podocarpus nubigenus* | 5 | 0 | 9 | 2 | 0 |
|  | *Amomyrtus meli* | 0 | 0 | 3 | 0 | 0 |
|  | *Mirceugenia* | 1 | 0 | 0 | 0 | 0 |
|  | *myrceugenia parviflora* | 0 | 0 | 1 | 0 | 1 |
|  | *Raphitmmus* | 2 | 0 | 1 | 0 | 0 |
|  | *Raukaua laetevirens* | 1 | 0 | 0 | 0 | 0 |
|  | *Laureliopsis phillippiana* | 1 | 2 | 3 | 0 | 0 |
|  | *Tepualia stipularis* | 5 | 0 | 9 | 0 | 0 |
|  | *Caldcluvia paniculata* | 3 | 4 | 12 | 0 | 0 |
|  | *Weinmannia trichosperma* | 1 | 0 | 1 | 1 | 0 |
|  | *Eucryphia cordifolia* | 1 | 0 | 4 | 0 | 0 |

1. QUILAR FOREST

|  |  | Epiphyte species | | | | |
| --- | --- | --- | --- | --- | --- | --- |
|  |  | *Miraria coccinea* | *Asteranthera ovata* | *Luzuriaga polyphylla* | *Sarmienta repens* | *Campsidium valdivianum* |
| Tree species | *Gevuina avellana* | 2 | 0 | 4 | 0 | 0 |
|  | *Drymis winteri* | 16 | 1 | 38 | 0 | 1 |
|  | *Crinodendron hookerianum* | 0 | 1 | 6 | 1 | 1 |
|  | *Nothofagus dombeyi* | 16 | 2 | 27 | 0 | 1 |
|  | *Amomyrtus luma* | 26 | 3 | 47 | 3 | 1 |
|  | *Saxegothaea conspicua* | 1 | 1 | 4 | 1 | 2 |
|  | *Podocarpus nubigenus* | 2 | 2 | 12 | 0 | 0 |
|  | *Amomyrtus meli* | 1 | 0 | 1 | 0 | 0 |
|  | *myrceugenia parviflora* | 0 | 1 | 1 | 1 | 1 |
|  | *Raukaua laetevirens* | 6 | 0 | 3 | 0 | 0 |
|  | *Laureliopsis phillippiana* | 3 | 0 | 5 | 2 | 0 |
|  | *Tepualia stipularis* | 3 | 0 | 37 | 2 | 7 |
|  | *Caldcluvia paniculata* | 1 | 0 | 8 | 1 | 3 |
|  | *Eucryphia cordifolia* | 2 | 0 | 7 | 2 | 0 |
